# Prevalence of occupational stress-related syndromes among health care workers in Latin America from 2019 to 2023

**DOI:** 10.47626/1679-4435-2024-1329

**Published:** 2025-08-25

**Authors:** Lilian García-Pérez, Yadira Maria Pino, Elisa Ansoleaga

**Affiliations:** 1 Doctorate in Psychology, Faculty of Psychology, Universidad Diego Portales, Santiago, Metropolitan Region, Chile; 2 Independent investigator, Cape Coral, FL, USA; 3 Faculty of Psychology, Universidad Diego Portales, Santiago, Metropolitan Region, Chile

**Keywords:** health personnel, psychological stress, burnout, Latin America., personal de salud, estrés psicológico, burnout, Latinoamérica.

## Abstract

This study aimed to systematically map existing evidence on the prevalence of work-related stress syndromes in the health care sector in Latin America and their associations with sociodemographic and occupational variables, with the goal of identifying knowledge gaps. Relevant studies published between 2019 and 2023 were reviewed across three databases following the Preferred Reporting Items for Systematic Reviews and Meta-Analyses (PRISMA) guidelines for scoping reviews. Study characteristics, sample details, explored syndromes, and results were recorded. The search identified 7,898 articles. After removing duplicates and assessing eligibility criteria, 67 articles were reviewed, with 12 included in the final analysis. Brazil had the highest number of included studies. Most samples consisted of women and physicians and nurses. All studies addressed burnout syndrome, with reported prevalence ranging from 13.2% to 70.3%. High emotional exhaustion was reported in 15.9% to 39.4% of participants, high depersonalization in 11.8% to 44.2%, and low personal fulfillment in 0% to 61.3%. Findings on sociodemographic and occupational variables were heterogeneous, showing both convergence and divergence with existing literature. Information on work-related stress syndromes in Latin American health care professionals is largely limited to burnout, with a focus on mental health and workplace conditions within specific groups and professions. There is an urgent need to explore other syndromes and professional categories to identify additional factors affecting the mental health of this population.

## Abstract (es)

La presente investigación se propuso mapear sistemáticamente la evidencia existente sobre la prevalencia de síndromes relacionados con el estrés laboral en el sector salud en Latinoamérica y sus vínculos con variables sociodemográficas y laborales, con el fin de identificar brechas en el conocimiento. Siguiendo las directrices Preferred Reporting Items for Systematic Reviews and Meta-Analyses para revisiones de alcance, se revisaron en tres bases de datos estudios relevantes al tema publicados entre 2019-2023. Se registraron características del estudio, de las muestras, síndromes explorados y resultados obtenidos. La búsqueda identificó 7.898 artículos. Tras eliminar duplicados y evaluar según los criterios de elegibilidad, se revisaron 67 trabajos a texto completo, de los cuales 12 fueron incluidos en la revisión. El país con más estudios incluidos fue Brasil. La mayoría de las muestras estuvieron compuestas por mujeres y personal médico y de enfermería. Todos los estudios incluidos evaluaron el *burnout*. Se informan prevalencias del síndrome del 13,2 al 70,3%, con valores de alto cansancio emocional del 15,9 al 39,4%, alta despersonalización del 11,8 al 44,2%, y baja realización personal del 0 al 61,3%. El comportamiento de las variables sociodemográficas y laborales es heterogéneo y presenta puntos de encuentro y ruptura con la literatura. La información sobre la prevalencia de síndromes relacionados con el estrés laboral en personal sanitario en Latinoamérica está limitada al *burnout*, notándose enfoque en la relación entre salud mental y condiciones y organización del trabajo en grupos y profesiones específicas. Urge explorar otros síndromes y profesionales para identificar nuevos factores asociados a la salud mental de esta población.

## INTRODUCTION

The definition of stress is often associated with negative connotations. However, as Sapolsky^1^ (2015) noted, when understood as an appropriate response to perceived threats, whether real or potential, stress has significant adaptive potential. Its effects on the brain and behavior follow an inverted U-shaped pattern, establishing a link between stimulus intensity to response levels. While individual variability plays a role, certain levels of stress are necessary and beneficial for specific tasks, whereas excessive stress becomes harmful.^1^ Psychiatry has documented the effects of highly stressful situations, both acute and prolonged, examining a range of responses from transient reactions to trauma-related disorders.^2^

Therefore, stress is an inherent part of life. In this article, the term will specifically refer to harmful stress. Its chronicity in adulthood is known to impact social behavior, disrupt related brain circuits, and increase the risk of physical and mental illnesses.^3^

Stress has been studied in different specific settings, including the workplace. The World Health Organization (WHO)^4^ highlights the influence of work characteristics and conditions on its onset. The primary syndromes associated with work-related stress include burnout, vicarious trauma, compassion fatigue, and secondary traumatic stress.

Burnout syndrome is associated with a discrepancy between job demands and a worker’s ability to cope with them,^5^ and has been commonly studied in the caregiving settings. Etiologically, burnout is linked to work conditions and organizational factors and is characterized by emotional exhaustion, low personal fulfillment, and depersonalization.^6^ In other words, affected workers may become insensitive, cynical, and unmotivated, experiencing persistent physical and emotional exhaustion. Additionally, they may develop sleep and eating disorders, headaches, physical pain, and difficulty breathing, which can lead to absenteeism or job abandonment.^5,7^

Vicarious trauma is defined as a caregiver’s response to the trauma experienced by the person they are assisting. Its incidence has been primarily documented among therapists working with trauma victims.^8^ In this context, it manifests through cognitive symptoms similar to those of post-traumatic stress disorder (PTSD), such as distrust, pessimism, feelings of unsafety and lack of autonomy, intrusive thoughts, and memory problems.^9^

Compassion fatigue was first documented among nursing staff^10^ and manifests as a decrease in empathetic capacity, leading to a significant loss of the ability to provide adequate care. Fatigue, apathy, sadness, feelings of incapacity, and irritability are reported, along with physical symptoms similar to those of burnout. It is essential to emphasize the exhaustion caused by sustained empathy,^11^ distinguishing this syndrome from others by highlighting compassion and empathy as responses to high emotional demands at work. Compassion fatigue was initially identified in the health care sector, where affective labor is inherently linked to caregiving. This emotional work, often overlooked, adds to the burden of clinical tasks.^12^ There are theoretical divergences regarding its onset: while some authors suggest it develops gradually and cumulatively,^11^ others describe a sudden onset triggered by an intense new emotional demand.^13^

Secondary traumatic stress is a term that can be used as an alternative to compassion fatigue,^8^ and results from working with individuals who have experienced trauma. Its symptoms combine features of both burnout and PTSD, with a sudden onset in the professional, triggered by contact with a patient suffering from trauma.^14^ Due to theoretical and methodological challenges arising from the plurality of definitions, the terms have been used interchangeably in the literature.^13^ In this study, compassion fatigue will be interpreted as a cumulative process, while secondary traumatic stress as an acute reaction. The diversity of conditions and definitions has created challenges for research. The literature has identified conceptual and empirical overlaps between these syndromes and has made progress in systematizing this subject.^15^

A significant part of the scientific evidence on the topic focuses on health care professionals, whose work is often characterized by excessive workloads, high cognitive, emotional, and physical demands, difficulties in managing boundaries between personal and professional life, weakened work teams, lack of role clarity, and an imbalance between effort and reward.^16-19^

Following the COVID-19 pandemic, interest in health care professionals’ mental health has grown, leading to an increase in scientific research on the subject.^20^ A systematic review conducted during the pandemic, covering studies from North America, Europe, and Asia, reported alarming prevalence rates of mental health issues among health care personnel: post-traumatic stress (3-84%), anxiety (3-97%), depression (8-95%), and psychological distress (3-76%).^21^ Similarly, in 2022, the Pan American Health Organization (PAHO) reported significant rates of depressive symptoms (14.7-22%), psychological distress (12-13.5%), and suicidal ideation (5-15%) among health care workers in Latin America.^20^ Additionally, a qualitative study on these professionals identified work overload in both domestic and paid labor, fear of infection and transmission, sadness, loneliness, distress, uncertainty, and isolation.^22^

However, mental health issues in this sector predate the pandemic. A 2019 systematic review of studies from North America and Europe reported significant prevalence rates of burnout (18.2-28.4%), secondary traumatic stress (16.6-25.8%), and compassion fatigue (41.8-43.3%).^23^ Another review found differences in burnout levels between physicians and nurses. Among physicians, emotional exhaustion ranged from 21% to 45%, high depersonalization from 25% to 71%, and low personal fulfillment from 25% to 70%. Among nurses, emotional exhaustion ranged from 28% to 31%, high depersonalization from 15% to 24%, and low personal fulfillment from 25% to 70%.^24^

Conversely, in 2018, an umbrella review found a significant prevalence of burnout in Latin America, with notable rates of emotional exhaustion (29.3-30.8%), depersonalization (20.6-29.3%), and low personal fulfillment (73.1-83.9%). The study highlights the influence of cultural and linguistic factors in analyzing this issue, as these differences may affect the results.^25^

Sociodemographic and organizational variables influence the onset and manifestation of these syndromes and other mental health conditions in this population. Factors linked to burnout include profession, age, education, experience, sex, work shifts, workload, and type of institution, among others.^26-31^ The health care workforce is predominantly composed of women^32^; however, the association between burnout and gender remains unclear in the literature. Some studies report higher rates of the syndrome in women, while others find no significant differences.^33^ Regarding age, younger workers are more likely to experience burnout.^34^ However, the literature is inconclusive about the influence of professional experience, with some studies suggesting that less experience increases the likelihood of burnout,^31^ while others find no relationship between the variables.^34^

Health care workers’ mental health is essential to their performance and, consequently, to the fulfillment of their social role. Most research on work-related stress, primarily conducted in North America and Europe, focuses on burnout syndrome among medical and nursing staff. However, working conditions in Latin America differ from those in the Global North, highlighting the need to examine the evidence in this specific setting.

This scoping review aims to describe and analyze the existing scientific evidence on the prevalence of work-related stress syndromes in the health care sector in Latin America and their associations with sociodemographic and work-related variables. Considering the contextual and theoretical background, this study seeks to answer the following questions: “What are the characteristics of the existing scientific evidence on the prevalence of work-related stress syndromes in the health care sector in Latin America?” and “What associations have been identified between these syndromes and the sociodemographic and work-related variables studied?”

## METHODS

A scoping review was conducted to describe how research on work-related stress syndromes in the health care sector in Latin America has been approached and to examine its findings. This descriptive study considered both quantitative and qualitative approaches. The review protocol was established following the Preferred Reporting Items for Systematic Reviews and Meta-Analyses extension for Scoping Reviews (PRISMA-ScR).^35^

### IDENTIFICATION OF RELEVANT STUDIES

The literature search was conducted in September and October of 2023 using the following databases: PubMed, Web of Science, and Scopus. The search terms included: “compassion fatigue” OR “secondary traumatic stress” OR “vicarious trauma” OR “burnout” AND “healthcare” OR “healthcare worker” OR “health worker” OR “healthcare professional” OR “health professional” OR “healthcare provider” OR “health provider” OR “healthcare personnel” OR “health personnel”.

The following inclusion criteria were established: 1. Empirical studies assessing the prevalence of work-related stress syndromes in institutionalized, actively employed, and salaried health care workers; 2. Studies conducted with Latin American populations; 3. Articles published in journals indexed in the selected databases; 4. Articles published in Spanish, English, or Portuguese. Search terms were in English, as titles and abstracts are often available in English even when the full text is in Spanish or Portuguese; 5. Articles published between January 1, 2019, and September 30, 2023, with data collected prior to the pandemic. This criterion aimed to exclude the impact of the health crisis and focus on the pre-pandemic context.

The exclusion criteria were as follows: 1. Articles in which the study population was not Latin American or in which its origin was unspecified; 2. Studies involving administrative workers and/or students in the health care sector, including those that did not provide a separate analysis for these groups. These participants were excluded due to their distinct working conditions, which differ from direct health care practice and could influence the results; 3. Studies that collected data during or after the pandemic or where the data collection period was unclear. When this information was missing, the authors were contacted.

### STUDY SELECTION

A database was created using EndNote version 20, compiling all the studies identified in the search. Duplicates were removed using automated tools, followed by a manual search. The remaining studies were exported to an Excel database. Two reviewers independently assessed the titles and abstracts based on the eligibility criteria. In cases of discrepancies, the full article was reviewed. Next, both reviewers read the full texts of the remaining articles. Two articles were excluded from the study after two unsuccessful attempts to contact the authors regarding data collection.

### DATA EXTRACTION

The analysis extracted relevant information from the articles using 11 categories, including study characteristics, sample details, sociodemographic and work-related factors, syndromes addressed, and results obtained. Two independent reviewers entered the data into an Excel matrix. Any discrepancies were resolved by consulting the original articles.

## RESULTS

**Figure 1:**
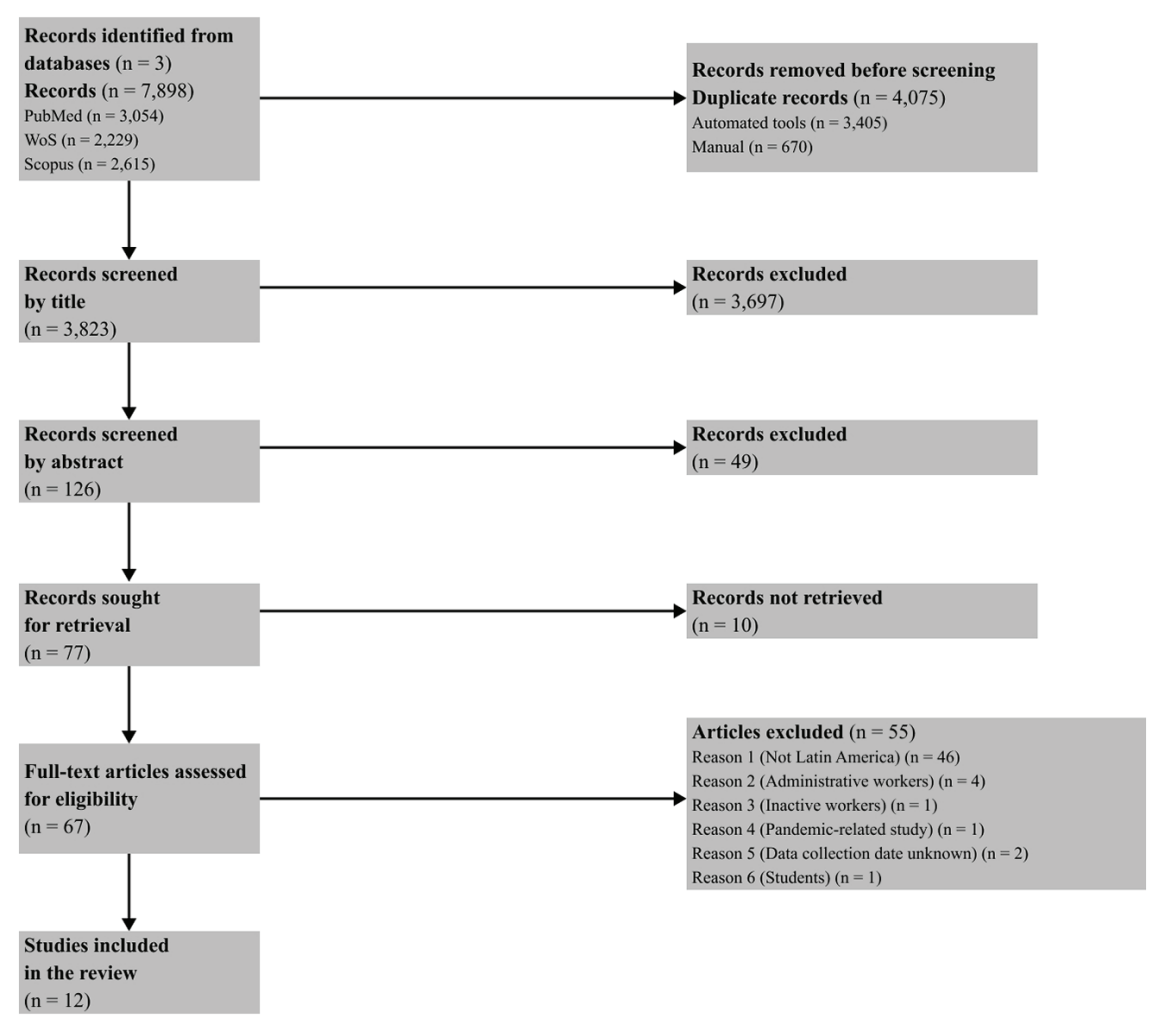
Preferred Reporting Items for Systematic Reviews and Meta-Analyses (PRISMA) flow diagram of the review process.

The database search yielded 7,898 results. After the review, 67 studies were fully examined, and 12 were included in the study. The process is illustrated in Figure 1.

Brazil had the highest number of included studies (33.3%), followed by Mexico (25%) and Colombia (16.7%). All articles were published in medical science journals. Most studies were in Spanish (58.3%), while the remaining were in English (41.7%). The most frequent publication years were 2021 (50%) and 2020 (33.3%) (Table 1). The studies included 5,635 participants, mostly women, with mean ages ranging from 33.7 to 45.1 years.

**Table 1:** Articles included in the scope review

Title	Authors	Journal	Year	Country	Language	Syndrome addressed
Síndrome de desgaste en profesionales de la salud mexicanos. Médicos: ¿mártires o víctimas de su profesión?	Romero-González et al.^37^	Medicina Interna de México	2021	Mexico	Spanish	Burnout
Síndrome de quemarse por el trabajo en atención de pacientes oncológicos	Ramírez-Pérez & Osorio-Guzmán^38^	Revista Médica del Instituto Mexicano de Seguro Social	2023	Mexico	Spanish	Burnout
Comportamiento del síndrome de burnout y la resiliencia en trabajadores de cuidados intensivos	Gutiérrez-Sánchez et al.^39^	Medisur	2021	Cuba	Spanish	Burnout
Burnout syndrome among healthcare professionals in intensive care units: a cross-sectional population-based study	Alvares et al.^40^	Revista Brasileira de Terapia Intensiva	2020	Brazil	English	Burnout
Síndrome de burnout en el personal de enfermería de unidades de cuidado crítico y de hospitalización	Rendón Montoya et al.^41^	Enfermería Global	2020	Mexico	Spanish	Burnout
Burnout among primary health care workers in Brazil: results of a multilevel analysis	Silva et al.^42^	International Archives of Occupational and Environmental Health	2021	Brazil	English	Burnout
Intervening variables of burnout in health professionals of emergency services	Pereira et al.^43^	Texto & Contexto Enfermagem	2021	Brazil	English	Burnout
Síndrome de burnout y ansiedad en médicos de la ciudad de Santa Marta	Bresó-Esteves et al.^44^	Duazary	2019	Colombia	Spanish	Burnout
Professional burnout syndrome in health professionals	Fajardo-Lazo et al.^45^	Archivos Venezolanos de Farmacologia y Terapeutica	2021	Ecuador	English	Burnout
Síndrome de burnout em profissionais de saúde atuantes na atenção básica: um estudo transversal	Frota et al.^46^	Journal of Physiotherapy Research	2021	Brazil	English	Burnout
Engagement y burnout en profesionales de la salud colombianos	Quiroz et al.^47^	Salud y Ciencia	2020	Colombia	Spanish	Burnout
Síndrome de desgaste profesional en trabajadores de la salud de una Unidad de Cuidados Intensivos en un hospital del Estado de Aragua, Venezuela	Rodríguez & Ortunio^48^	Comunidad y Salud	2020	Venezuela	Spanish	Burnout

Studies predominantly focused on medical and nursing personnel (Table 2). Although the search criteria allowed for the inclusion of qualitative studies, all included studies used a quantitative methodology with a cross-sectional observational design. In assessing methodological quality,^36^ most studies received a good evaluation, except for one, which was rated as moderate due to the use of a non-validated instrument and limited methodological description.^37^

**Table 2:** Distribution of sex, profession, and mean age in the samples of the included studies

Category	Participants	Number of studies (n = 12)	Percentage (%)
Sex	Majority men (> 50%)	1	8.3
	Majority women (> 50%)	10	83.3
	Equal men and women (50% each)	1	8.3
Profession	Physicians	1	8.3
	Nursing staff	2	16.7
	Physicians and nursing staff	6	50.0
	Physicians, nursing staff, and other professionals	2	16.7
	Other professionals	1	8.3
Age (years)	31-40	7	58.3
	41-50	2	16.7
	Not reported	3	25.0

All articles included in this review assessed burnout prevalence. The most commonly used instruments were the Maslach Burnout Inventory (MBI) (41.7%) and its version for human services, the MBI-Human Services Survey (MBI-HSS) (41.7%). Additionally, one study used a non-validated semi-structured survey,^37^ and another applied the Cuestionario para la Evaluación del Síndrome de Quemarse por el Trabajo (CESQT, Burnout Syndrome Evaluation Questionnaire) adapted for health care personnel.^38^ Most studies (66.7%) reported burnout prevalence, ranging from 13.2% to 70.3% (Table 3).^37-44^ The remaining studies presented results based exclusively on burnout dimensions.^45-48^ High emotional exhaustion ranged from 15.9% to 39.4%, high depersonalization from 11.8% to 44.2%, and low personal fulfillment from 0% to 61.3% (Table 3). Only one study referred to physiological symptoms, reporting neck pain, lower back pain, and pain or heaviness in the lower limbs.^48^

**Table 3:** Distribution of burnout indices and their dimensions in the included studies

Title	Authors	Number of participants (n)	Burnout prevalence (%)	High emotional exhaustion (%)	High depersonalization (%)	Low personal accomplishment (%)
1- Síndrome de burnout y ansiedad en médicos de la ciudad de Santa Marta	Bresó-Esteves et al.^44^	59	25.4	N/R	N/R	N/R
2- Burnout syndrome among healthcare professionals in intensive care units: a cross-sectional population-based study	Alvares et al.^40^	241	43.4	26.4	15.2	10.2
3- Síndrome de desgaste profesional en trabajadores de la salud de una Unidad de Cuidados Intensivos en un hospital del Estado de Aragua, Venezuela	Rodríguez & Ortunio^48^	33	N/R	N/R	N/R	50.0
4- Síndrome de burnout en el personal de enfermería de unidades de cuidado crítico y de hospitalización	Rendón Montoya et al.^41^	90	56.7	18.9	21.1	28.9
5- Engagement y burnout en profesionales de la salud colombianos	Quiroz González et al.^47^	388	N/R	N/R	N/R	N/R
6- Burnout among primary health care workers in Brazil: results of a multilevel analysis	Silva et al.^42^	2,940	11.4	39.4	11.8	18.3
7- Professional burnout syndrome in health professionals	Fajardo-Lazo et al.^45^	208	N/R	15.9	44.2	23.6
8- Síndrome de burnout em profissionais de saúde atuantes na atenção básica: um estudo transversal	Frota et al.^46^	13	N/R	23.1	15.4	0.0
9- Intervening variables of burnout in health professionals of emergency services	Pereira et al.^43^	282	13.2	30.5	25.5	61.3
10- Síndrome de desgaste en profesionales de la salud mexicanos. Médicos: ¿mártires o víctimas de su profesión?	Romero-González et al.^37^	710	49.6	N/R	N/R	N/R
11- Comportamiento del síndrome de burnout y la resiliencia en trabajadores de cuidados intensivos	Gutiérrez-Sánchez et al.^39^	74	70.3	N/R	N/R	N/R
12- Síndrome de quemarse por el trabajo en atención de pacientes oncológicos	Ramírez-Pérez & Osorio-Guzmán^38^	41	19.5	N/R	N/R	N/R

N/R = not reported.

Some studies (25%) reported distinct results by profession. Burnout levels were higher in physicians compared to other health care workers.^41,47^ Working in pediatric intensive care units,^40^ oncology services,^38^ geriatrics, and internal medicine^37^ was associated with higher emotional exhaustion and burnout rates. Burnout prevalence was also higher among public institutions workers compared to private sector staff,^47^ as well as among professionals working in both sectors.^37^ Factors linked to severe burnout included lack of supervisory support, working with underprivileged populations, and poor workplace infrastructure.^42^ Higher work experience was also associated with increased burnout prevalence^38^; however, this association was not observed in another study.^41^

In nursing staff, working in general hospital wards was associated with higher emotional exhaustion compared to those working with critically ill patients. A sense of vocation for the service and taking breaks during work shifts were linked to higher personal satisfaction. Taking vacations once or twice a year was inversely related to burnout, while working morning, afternoon, and night shifts was associated with higher burnout levels.^41^

Regarding sociodemographic variables, age was inversely related to high levels of burnout.^40,42^ Being Black was also associated with a higher prevalence of severe burnout.^42^ One study assessed the link between gender and burnout, but no statistically significant relationships were found.^41^ Additionally, burnout was associated with the following variables: education, early stress, lifestyle, anxiety, and depression.^43^

## DISCUSSION

The present scoping review explored scientific evidence from Latin America on work-related stress syndromes among health care workers. Although qualitative studies were intended for inclusion, all the relevant publications used quantitative methodologies. This underscores the need for a qualitative approach to better understand the phenomenon through comprehensive and in-depth examination. All the included studies focused on burnout prevalence, highlighting its dominance over other stress syndromes and reinforcing the previously identified knowledge gap. This limitation may stem from theoretical and methodological overlaps in addressing these syndromes, as discussed by Wynn.^15^

As described by García-Arroyo & Osca Segovia,^25^ burnout syndrome is a prevalent issue in Latin America. However, the results and their analysis are significantly heterogeneous due to differences in the instruments, procedures, and diagnostic criteria used. This presents a challenge in systematizing knowledge and underscores the need for standardization in future research. Conversely, the predominance of women and medical and nursing personnel in the samples is consistent with previous studies,^32^ highlighting the need to expand research to include other professions and provide an intersectional perspective, especially considering the experiences during the pandemic.

Regarding sociodemographic and work-related variables, the results also show heterogeneity due to the diversity of the elements evaluated and the ways they are interpreted. Regarding gender and age, the data is consistent with the literature, as no statistically significant associations were found between gender and burnout, while older age was identified as a protective factor.^31,33,43^

It is noteworthy that, in one study, greater work experience (typically associated with older age) was related to a higher likelihood of experiencing burnout.^38^ Therefore, this review was unable to draw conclusions about the relationship between age, work experience, and burnout, as found in the literature.^34^ This highlights the need for further investigation into how gender, age, and burnout are related and how they mediate each other. Additionally, consistent with the literature, the following trends are evident: the influence of the type of institution, with higher burnout prevalence observed among professionals in the public sector; differences between medical staff, nursing staff, and other health care professionals; variations across different medical services; higher burnout levels in morning, evening, and night shifts; and associations with other mental health conditions, such as anxiety and depression.^26-29,31^

This scoping review has some limitations. Firstly, it is possible that not all relevant studies were identified, as some may have been published in other databases, grey literature, or may not be publicly available. The exclusion of certain worker groups, such as administrative staff, students, non-institutionalized workers, and volunteers, also limits the scope of the analysis. Future scoping reviews could include these groups to broaden the perspective on the topic.

Despite these limitations, this review provides an overview of the current state of research on work-related stress syndromes among health care workers in Latin America. It is noteworthy that, although a significant portion of the evidence focuses on the pandemic period,^20,21^ the issue extends beyond that, as it predates the COVID-19 pandemic and remains highly prevalent in the region. Furthermore, the complexities in addressing and interpreting results based on sociodemographic and work-related variables are significant, as they are influenced by the region’s cultural and research diversity.

The predominance of studies on burnout syndrome in the region emphasizes the need to analyze the association between mental health and working conditions. This raises the question of how emotional demands impact the mental health of health care professionals, which could be explored through the study of syndromes such as compassion fatigue. From this approach, knowledge gaps are identified, providing a foundation for future research and policy development.

## CONCLUSIONS

Stress is an inevitable aspect of work for health care professionals, making it essential to address its consequences, not only for their well-being but also for society in general, as it constitutes a public health issue. The present scoping review has emphasized the predominance of burnout-focused studies, whose findings and labor and sociodemographic composition show both convergences and divergences with the literature. Links between burnout and sociodemographic and work-related variables were confirmed, though discrepancies were noted regarding the association between work experience and burnout. The need for standardization in the identification and presentation of results is emphasized, as it would contribute to the systematization of knowledge. Furthermore, this review highlights the importance of expanding research into other syndromes that, through their conceptualization, could uncover new elements affecting the mental health of this population. It also calls for broadening studies to include other professions and countries from an intersectional perspective, taking cultural factors into account. These aspects are key to advancing the understanding and management of this issue.
